# Severe and fatal adverse events of immune checkpoint inhibitor combination therapy in patients with metastatic renal cell carcinoma: a systematic review and meta-analysis

**DOI:** 10.3389/fimmu.2023.1196793

**Published:** 2023-06-19

**Authors:** Yao-Ning Feng, Guang-Yu Xie, Li Xiao, Dun-Chang Mo, Jian-Feng Huang, Peng-Hui Luo, Xiu-Juan Liang

**Affiliations:** ^1^Urology Surgery Department, The Fifth Affiliated Hospital of Guangxi Medical University, Nanning, Guangxi, China; ^2^Department of Critical Care Medicine, The Fifth Affiliated Hospital of Guangxi Medical University, Nanning, Guangxi, China; ^3^Radiotherapy Department, The Third Affiliated Hospital of Guangxi Medical University, Nanning, Guangxi, China

**Keywords:** renal cell carcinoma, tyrosine kinase inhibitor, immune checkpoint inhibitor, serious adverse events, fatal adverse events

## Abstract

**Introduction:**

Immune checkpoint inhibitor (ICI) combination therapy has changed the treatment landscape for metastatic renal cell carcinoma (mRCC). However, little evidence exists on the treatment-related severe adverse events (SAEs) and fatal adverse events (FAEs) of ICI combination therapy in mRCC.

**Method:**

We searched PubMed, Embase, and Cochrane Library databases to evaluate randomized controlled trials (RCTs) of ICI combination therapy versus conventional tyrosine kinase inhibitor (TKI)-targeted therapy in mRCC. Data on SAEs and FAEs were analyzed using revman5.4 software.

**Results:**

Eight RCTs (n=5380) were identified. The analysis showed no differences in SAEs (60.5% vs. 64.5%) and FAEs (1.2% vs. 0.8%) between the ICI and TKI groups (odds ratio [OR], 0.83; 95%CI 0.58−1.19, p=0.300 and OR, 1.54; 95%CI 0.89−2.69, p=0.120, respectively). ICI-combination therapy was associated with less risk of hematotoxicities, including anemia (OR, 0.24, 95%CI 0.15–0.38, p<0.001), neutropenia (OR, 0.07, 95%CI 0.03–0.14, p<0.001), and thrombocytopenia (OR, 0.05, 95%CI 0.02−0.12, p<0.001), but with increased risks of hepatotoxicities (ALT increase [OR, 3.39, 95%CI 2.39–4.81, p<0.001] and AST increase [OR, 2.71, 95%CI 1.81−4.07, p<0.001]), gastrointestinal toxicities (amylase level increase [OR, 2.32, 95%CI 1.33–4.05, p=0.003] and decreased appetite [OR, 1.77, 95%CI 1.08–2.92, p=0.020]), endocrine toxicity (adrenal insufficiency [OR, 11.27, 95%CI 1.55–81.87, p=0.020]) and nephrotoxicity of proteinuria (OR, 2.21, 95%CI 1.06−4.61, p=0.030).

**Conclusions:**

Compared with TKI, ICI combination therapy has less hematotoxicity in mRCC but more specific hepatotoxicity, gastrointestinal toxicity, endocrine toxicity, and nephrotoxicity, with a similar severe toxicity profile.

**Systematic review registration:**

https://www.crd.york.ac.uk/prospero/, identifier CRD42023412669.

## Introduction

Kidney cancer is one of the most common and dangerous cancer killers worldwide, and its incidence increases annually (1). Clear Cell renal cell carcinoma (RCC) accounts for approximately 90% of all kidney cancer pathologies (2). For decades, highly effective treatments for metastatic RCC (mRCC) have been lacking, and patients’ prognoses are very poor (3, 4). Vascular endothelial growth factor (VEGF)-tyrosine kinase inhibitor (TKI)-targeted therapies, such as sunitinib and cabozantinib, have certain efficacy and are recommended by international guidelines as the first-line treatment for mRCC; however, their overall treatment effect is not ideal, with a 5-year overall survival rate of less than 10% (5, 6). Moreover, VEGF-TKI–targeted agents have very common and unique adverse drug reactions that add pain to patients, including hypertension, proteinuria, hemorrhagic disease, and hand and foot syndrome (7).

In recent years, researchers have found that tumor cells can inhibit the immune function of T cells by promoting the activation of immune checkpoint molecules, thus avoiding being killed by the human immune system (8). With the development of immunotherapies and the approved application of immune checkpoint inhibitors (ICIs), ICI-based therapies have become promising treatment strategies for mRCC (9). Because of the widespread use of ICIs, immune-related adverse events have attracted considerable clinical attention. ICI immunotherapy is reported to cause multiple organ toxicities, including skin toxicity, gastrointestinal toxicity, liver toxicity, cardiovascular toxicity, and hematological toxicity. Some serious adverse events (SAEs) have a low incidence, including immune-associated pneumonia, myocarditis, and hepatitis; however, once an SAE occurs, it can be fatal (10, 11).

Combination therapies with ICIs have been widely used to treat mRCC. However, whether this combination strategy increases drug-related toxicity, particularly severe and fatal adverse events (FAEs), remains unclear. To explore the safety profile of ICI combination therapy and provide guidance for clinical treatment with ICIs, we conducted this meta-analysis comparing the SAEs and FAEs of ICI combination therapy with those of standard-of-care TKI-targeted therapy in patients with mRCC.

## Methods

### Search strategy

We searched the online electronic databases PubMed, Embase, and the Cochrane Library from inception until January 2023. The search keywords were *kidney cancer*, *renal cell carcinoma*, *renal cancer*, *immunotherapy*, *immune checkpoint inhibitors*, *PD-1*, *PD-L1*, *CTLA-4*, *nivolumab*, *pembrolizumab*, *camrelizumab*, *sintilimab*, *toripalimab*, *atezolizumab*, *avelumab*, *durvalumab*, *tremelimumab*, and *ipilimumab*. Only published randomized controlled trials (RCTs) were included; the languages were restricted to English.

### Literature inclusion and exclusion criteria

We included only the studies that met the following inclusion criteria (1): *Participants*: patients with metastatic, pathological, or histologically confirmed RCC (2); *Interventions*: Combination therapy with ICIs (e.g., ICI in combination with chemotherapy, targeted therapy, or endocrine therapy) (3); *Comparison*: TKI-targeted therapy (4); *Outcome*: at least one safety outcome for SAEs or FAEs was reported. The exclusion criteria included non-randomized controlled studies, retrospective studies, preclinical studies, animal studies, conference abstracts, reviews, case reports, letters, expert consensus literature, comments, articles with fewer than 10 patients, and incomplete data.

### Data extraction

The literature was screened independently by two researchers (FYN and XL), and relevant data were extracted and recorded using a standardized data collection form. Disagreements were resolved by a third independent researcher (MDC), and the final data were reviewed. Data were extracted from each study, including the study name, author information, year of publication, trial design, stage, number of patients, sex, age, treatment regimen, drug dose, and a detailed record of treatment-related SAEs and FAEs information. The SAEs were defined as Grade 3 or greater adverse events. FAEs were defined as Grade 5 adverse events (treatment-related deaths).

### Risk of bias and quality assessments

Two independent researchers (HJF and LPH) assessed bias in the selected studies using the Cochrane systematic risk of bias instrument (12), including random sequence generation, allocation concealment, blinding, blindness of outcome assessment, completeness of outcome data, reporting of selective outcomes, and other deviations. Any disagreements during the evaluation were resolved through discussion and consultation with an additional researcher (LXJ).

### Statistical analysis

The meta-analysis was performed using revman5.4 software. Differences were considered statistically significant at p<0.05. We used the odds ratio (OR) and 95% confidence interval (CI) for the risk assessment of SAEs and FAEs. Interstudy heterogeneity was determined using the Cochran Q and I^2^ statistical tests. Heterogeneity was assessed as *high* when I^2^ was >50% and *low* when I^2^ was <50%. A fixed-effects model was used when the statistical homogeneity was achieved (I^2^<50%). When statistical heterogeneity occurred between studies (I^2^>50%), a random-effects model was used for analysis.

## Results

### Search results and literature characteristics

**Figure 1:**
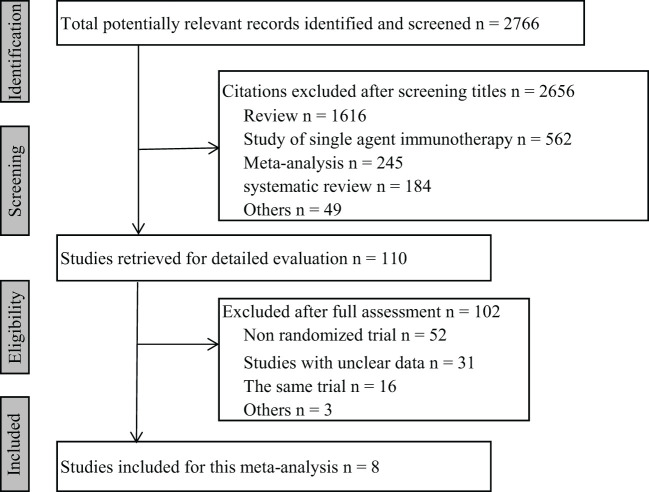
Flow diagram of the literature search.

After searching the databases, 2766 articles were retrieved. After screening and removing duplicates carefully, 8 RCTs comprising 5380 total patients with previously untreated mRCC were included in the analysis (**Figure 1**). Among the comparisons with TKI were 3 PD-1 plus VEGF inhibitors (pembrolizumab plus lenvatinib [13], nivolumab plus cabozantinib [14], and pembrolizumab plus axitinib [15]), 3 PD-L1 plus VEGF inhibitors (2 atezolizumab plus bevacizumab [16,18] and 1 avelumab plus axitinib [17]), and 2 PD-L1 plus CTLA-4 inhibitors (both nivolumab plus ipilimumab [19,20]). Among the 8 included trials, 6 were Phase 3 clinical studies and 2 were Phase 2. The selected studies were published between 2018 and 2022. The detailed characteristics of the included studies are shown in **Table 1**.

**Table 1:** The main characteristics of included studies.

Study	Study design	Phase	Sample size		Age, median (range)	Interventions	Median follow-up (months)
			Arms	N			
1. PD-1 plus VEGF inhibitors							
Motzer2021 (13) (CLEAR)	RCT	3	Study	352	64 (34-88)	Pembrolizumab (200 mg) intravenously every 3 weeks plus lenvatinib (20mg) orally once daily	26.6
			Control	340	61 (29-82)	Sunitinib (50mg) orally once daily for 4 weeks (6-weeks cycle)	
Choueiri2021 (14) (CheckMate 9ER)	RCT	3	Study	320	62 (29-90)	Nivolumab (240mg) intravenously every 2 weeks plus cabozantinib (40mg) orally once daily	18.1
			Control	320	61 (28-86)	Sunitinib(50mg) orally once daily for 4 weeks (6-weeks cycle)	
Thomas 2020 (15) (KEYNOTE-426)	RCT	3	Study	429	62 (55-68)	Pembrolizumab (200mg) intravenously every 3 weeks for up to 35 cycles plus axitinib (5mg) orally twice daily	30.6
			Control	425	61 (53-68)	Sunitinib (50mg) orally once daily for 4 weeks (6-weeks cycle)	
2. PD-L1 plus VEGF inhibitors							
Motzer2022 (16) (IMmotion151)	RCT	3	Study	451	62 (56-69)	Atezolizumab (1200mg) intravenously every 3 weeks plus bevacizumab (15mg/kg) intravenously every 3 weeks	36.1
			Control	446	60 (54-66)	Sunitinib (50mg) orally once daily for 4 weeks (6-weeks cycle)	
Motzer2019 (17) (JAVELIN Renal 101)	RCT	3	Study	434	62 (29-83)	Avelumab (10mg/kg) every 2 weeks plus axitinib(5mg) orally twice daily	19.3
			Control	439	61 (27-88)	Sunitinib (50mg) orally once daily for 4 weeks (6-weeks cycle)	
McDermott2018 (18) (IMmotion150)	RCT	2	Study	101	62 (32-88)	Atezolizumab (1200 mg) fixed intravenous dose plus bevacizumab 15 mg/kg every three weeks	20.7
			Control	100	61 (25-85)	Sunitinib (50 mg) orally once daily for 4 weeks (6-week cycle)	
3. PD-1 plus CTLA4 inhibitors							
Motzer2019 (19) (CheckMate 214)	RCT	3	Study	547	62 (26-85)	Nivolumab (3mg/kg) intravenously plus ipilimumab (1mg/kg) intravenously every 3 weeks for four doses, then nivolumab (3mg/kg) intravenously every 2 weeks	32.4
			Control	535	61 (21-85)	Sunitinib (50mg) orally once daily for 4 weeks(6-weeks cycle)	
Vano2022 (20) (BIONIKK)	RCT	2	Study	101	64 (49-73)	nivolumab 3 mg/kg intravenously plus ipilimumab 1 mg/kg every 3 weeks for four doses followed by intravenous nivolumab 240 mg every 2 weeks	18.0
			Control	40	65 (55-71)	sunitinib 50 mg/day for 4 weeks every 6 weeks or oral pazopanib 800 mg daily continuously	

RCT, randomized randomized trials; PD-1, programmed cell death protein-1; PD-L1, programmed cell death 1 ligand 1; VEGF, vascular endothelial growth factor; CTLA-4, cytotoxic T lymphocyte antigen 4.

### SAEs

**Figure 2:**
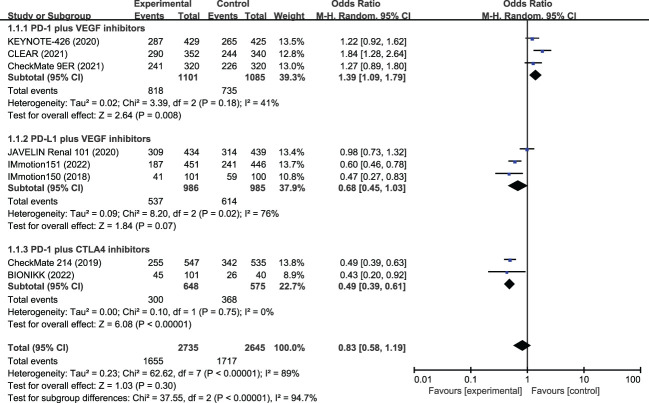
Meta-analysis of severe adverse events of immune checkpoint inhibitor combination therapy versus tyrosine kinase inhibitors in metastatic renal cell carcinoma. PD-1, programmed cell death protein-1; PD-L1, programmed cell death 1 ligand 1; VEGF, vascular endothelial growth factor; CTLA-4, cytotoxic T lymphocyte antigen 4.

**Figure 3:**
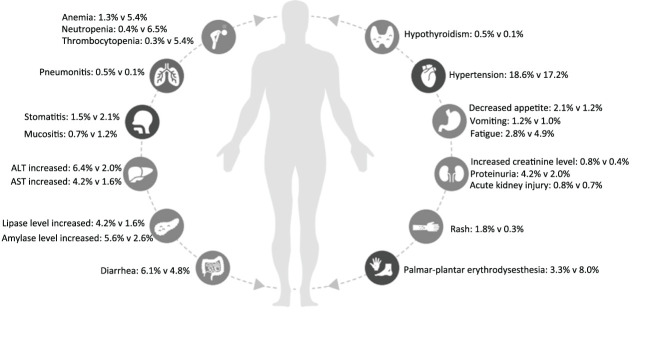
Severe adverse events of immune checkpoint inhibitor combination therapy versus tyrosine kinase inhibitors in each organ system for patients with metastatic renal cell carcinoma.

In the eight included studies, the incidence of SAEs was 60.5% in the ICI combination group (n=2735) and 64.9% in the TKI group (n=2645) (**Table 2**). As shown in **Figure 2**, the meta-analysis found no statistical difference in the risk of SAEs between the two treatment groups (OR, 0.83, 95%CI 0.58−1.19, p=0.300). Subgroup analysis showed that, compared with TKI alone, PD-1 plus CTLA-4 inhibitors had a lower risk of SAEs (OR, 0.49, 95%CI 0.39−0.61, p<0.001), while PD-1 plus VEGF inhibitors had a greater risk of SAEs (OR, 1.39, 95% CI 1.09−1.79, p=0.008). In the PD-L1 plus VEGF inhibitors subgroup, no difference in SAEs was found between the two treatment groups (OR, 0.68, 95%CI 0.45−1.03, p=0.070). The most common SAEs in the ICI combination group were hypertension (18.6%), increased lipase level (8.9%), increased ALT level (6.6%), diarrhea (6.1%), and increased amylase level (5.6%); the incidence of these corresponding SAEs was 17.2%, 6.6%, 2.0%, 4.8%, and 2.6% in the TKI group, respectively (**Table 2**; **Figure 3**). The meta-analysis showed that ICI-combination therapy had significantly less risk of hematotoxicities, including anemia (OR, 0.24, 95%CI 0.15–0.38, p<0.001), neutropenia (OR, 0.07, 95%CI 0.03–0.14, p<0.001), and thrombocytopenia (OR, 0.05, 95%CI 0.02−0.12, p<0.001) than TKI. However, for digestive toxicity and nephrotoxicity, ICI-combination therapy was associated with significantly greater risks of an ALT increase (OR, 3.39, 95%CI 2.39–4.81, p<0.001), AST increase (OR, 2.71, 95%CI 1.81−4.07, p<0.001), amylase level increase (OR, 2.32, 95%CI 1.33–4.05, p=0.003), decreased appetite (OR, 1.77, 95%CI 1.08–2.92, p=0.020), and proteinuria (OR, 2.21, 95% CI 1.06−4.61, p=0.030). In addition, greater risks of rash (OR, 5.01; 95%CI 2.11–11.9, p=0.003) and adrenal insufficiency (OR, 11.27; 95%CI 1.55–81.87, p=0.020) and less risk of mucosal inflammation (OR, 0.37; 95%CI 0.19–0.69, p=0.002) were observed with ICI combination therapy (**Table 2**).

**Table 2:** Severe adverse events of ICI combination therapy versus TKI.

	Event	Number of studies	ICI arm	TKI arm	OR (95% CI)	Effect	
			Pts with SAEs/total Pts, (%)	Pts with SAEs/total Pts, (%)		I ^2^ (%)	P value
	Total	8	1655/2735 (60.5)	1717/2645 (64.9)	0.83 (0.58-1.19)	89	0.30
Skin	Rash	5	32/1754 (1.8)	5/1674 (0.3)	5.01 (2.11-11.90)	35	0.0003
	Palmar-plantar erythrodysesthesia	7	88/2643 (3.3)	168/2099 (8.0)	0.38 (0.14-1.03)	88	0.06
	Mucosal inflammation	5	12/1735 (0.7)	34/1730 (1.2)	0.37 (0.19-0.69)	32	0.002
	Stomatitis	6	32/2087 (1.5)	43/2070 (2.1)	0.73 (0.46-1.16)	31	0.19
Hematotoxicity	Anemia	5	21/1636 (1.3)	84/1564 (5.4)	0.24 (0.15-0.38)	26	< 0.00001
	Decreased platelet count	5	7/1636 (0.4)	90/1564 (5.8)	0.09 (0.05-0.20)	0	< 0.00001
	Neutropenia	5	7/1636 (0.4)	101/1564 (6.5)	0.07 (0.03-0.14)	0	< 0.00001
	Thrombocytopenia	5	5/1986 (0.3)	107/1970 (5.4)	0.05 (0.02-0.12)	0	< 0.00001
Endocrine	Hypertension	7	406/2188 (18.6)	363/2110 (17.2)	1.04 (0.70-1.56)	78	0.84
	Hypothyroidism	6	12/2183 (0.5)	3/2099 (0.1)	2.63 (0.91-7.56)	0	0.07
	Fatigue	7	61/2188 (2.8)	103/2110 (4.9)	0.57 (0.41-0.78)	45	0.0006
	Nausea	4	18/1666 (1.1)	14/1650 (0.8)	1.28 (0.63-2.58)	29	0.49
	Adrenal insufficiency	2	14/648 (2.2)	0/575 (0)	11.27 (1.55-81.87)	4	0.02
Gastrointestinal	Diarrhea	8	167/2735 (6.1)	126/2645 (4.8)	1.14 (0.68-1.92)	74	0.62
	Decreased appetite	6	43/2087 (2.1)	24/2070 (1.2)	1.77 (1.08-2.92)	46	0.02
	Vomiting	5	23/1968 (1.2)	19/1970 (1.0)	1.02 (0.33-3.14)	53	0.98
	Increased lipase level	3	69/773 (8.9)	46/700 (6.6)	1.46 (0.99-2.17)	0	0.06
	Increased amylase level	3	43/773 (5.6)	18/700 (2.6)	2.32 (1.33-4.05)	25	0.003
Hepatic	Increased ALT level	6	139/2183 (6.4)	42/2099 (2.0)	3.39 (2.39-4.81)	0	< 0.00001
	Increased AST level	5	88/2082 (4.2)	33/2059 (1.6)	2.71 (1.81-4.07)	0	< 0.00001
Renal	Increased blood creatinine level	4	10/1320 (0.8)	5/1235 (0.4)	1.70 (0.60-4.81)	0	0.32
	Proteinuria	5	70/1653 (4.2)	32/1631 (2.0)	2.21 (1.06-4.61)	56	0.03
	Acute kidney injury	2	5/648 (0.8)	4/575 (0.7)	1.01 (0.27-3.77)	0	0.98
Pulmonary	Pneumonitis	6	12/2183 (0.5)	3/2099 (0.1)	2.27 (0.84-6.15)	0	0.11

ICI, immune checkpoint inhibitor; TKI, tyrosine kinase inhibitor; SAEs, severe adverse events; Pts, patients; OR, odds ratio.

### FAEs

**Figure 4:**
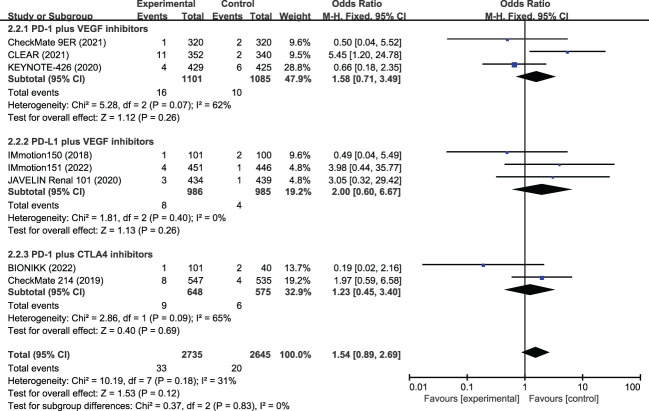
Meta-analysis of fatal adverse events of immune checkpoint inhibitor combination therapy versus tyrosine kinase inhibitor in metastatic renal cell carcinoma. PD-1, programmed cell death protein-1; PD-L1, programmed cell death 1 ligand 1; VEGF, vascular endothelial growth factor; CTLA-4, cytotoxic T lymphocyte antigen 4.

Data on FAEs were reported in all included studies. The most common FAEs in the ICI-combination group (n=2735) were pneumonitis (4 patients), hepatitis (3 patients), hemorrhagic events (3 patients), sepsis (3 patients), myocarditis (2 patients), myasthenic syndrome (2 patients), and sudden death (2 patients). In the TKI group (n=2645), they were heart failure (2 patients), pneumonia (2 patients), gastrointestinal hemorrhage (2 patients), and cardiac arrest (2 patients). The incidences of FAEs were 1.2% and 0.8% in the ICI and TKI combination groups, respectively (**Table 3**). The meta-analysis suggested the incidence of FAEs had no statistical difference (OR, 1.54, 95%CI 0.89−2.69, p=0.830) between the two treatment groups (**Figure 4**). Subgroup analysis showed that a similar risk of FAEs was found for PD-1 plus VEGR inhibitors (OR, 1.58, 95%CI 0.71−3.49, p=0.260), PD-L1 plus VGFR inhibitors (OR, 2.00, 95%CI 0.60−6.67, p=0.260), and PD-L1 with CTLA-4 inhibitors (OR, 1.23, 95%CI 0.45−3.40, p=0.690).

**Table 3:** Fatal adverse events of ICI combination therapy versus TKI.

Study	Number of treatment-related deaths/Total Pts, (%)		Treatment-related FAEs, (n)	
	ICI arm	TKI arm	ICI arm	TKI arm
Motzer2021 (13) (CLEAR)	11/352 (3.1)	2/340 (0.6)	acute renal failure (1), uncontrolled hypertension (1), myasthenic syndrome (1), autoimmune hepatitis (1), cardiac arrest (1), death-cause not specified (1); hemorrhagic events (2) , and sepsis (3)	respiratory failure and acute kidney injury (1) and death-cause not specified (1)
Choueiri2021 (14) (CheckMate 9ER)	1/320 (0.3)	2/320 (0.6)	small-intestine perforation (1)	pneumonia (1) and respiratory distress (1)
Thomas 2020 (15) (KEYNOTE-426)	4/429 (0.9)	6/425 (1.4)	myasthenia gravis (1), myocarditis (1), necrotising (1), fasciitis (1), and pneumonitis (1)	acute myocardial infarction (1), cardiac arrest (1), fulminant hepatitis (1), gastrointestinal haemorrhage (1), intracranial haemorrhage (1), and pneumonitis (1)
Motzer2022 (16) (IMmotion151)	4/451 (0.9)	1/446 (0.2)	NA	NA
Motzer2019 (17) (JAVELIN Renal 101)	3/434 (0.7)	1/439 (0.2)	sudden death (1), myocarditis (1), and necrotizing pancreatitis (1)	intestinal perforation (1)
McDermott2018 (18) (IMmotion150)	1/101 (0.9)	2/100 (2)	hemorrhage (1)	sudden death (1) and intestinal hemorrhage (1)
Motzer2019 (19) (CheckMate 214)	8/547 (1.5)	4/535 (0.7)	pneumonitis (3), aplastic anaemia (1), immune-mediated bronchitis (1), lower gastrointestinal haemorrhage (1), haemophagocytic syndrome (1), sudden death (1), and hepatitis (1)	cardiac arrests (2), heart failure (1), and multiple organ failure (1)
Vano2022 (20) (BIONIKK)	1/101 (0.9)	2/40 (5)	fulminant hepatitis (1)	heart failure (1 ) and thrombotic microangiopathy (1)
Total	33/2735 (1.2)	20/2645 (0.8)		

ICI, immune checkpoint inhibitor; TKI, tyrosine kinase inhibitor; FAEs, fatal severe adverse events; Pts, patients; NA, not, available.

### Quality of the included studies

**Figure 5:**
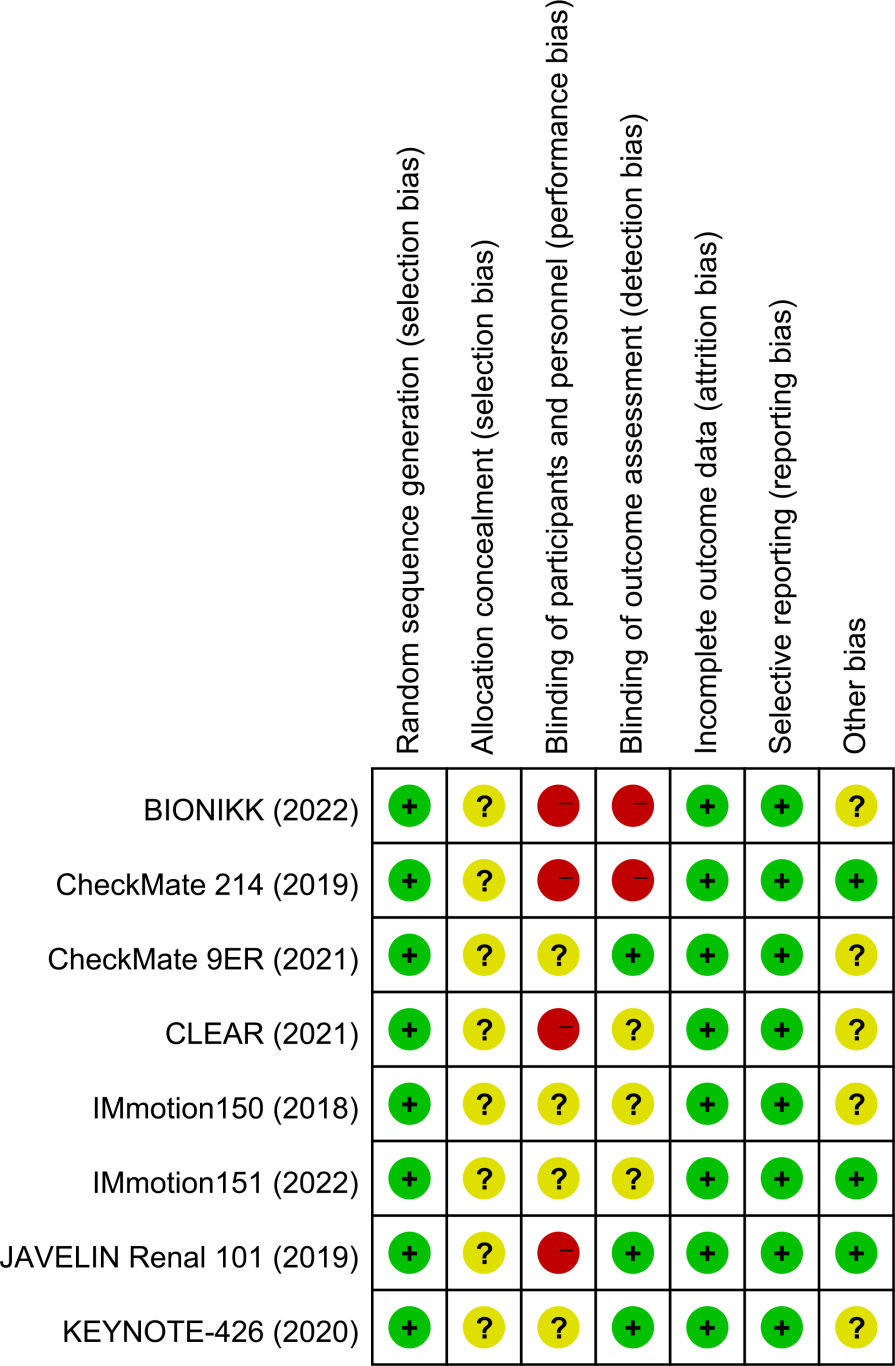
Quality evaluation of the included randomized controlled trials.

All eight included RCTs were assessed as *high quality* using the Cochrane Collaboration tool. The details are shown in **Figure 5**.

## Discussion

For over a decade, immunotherapy with ICIs has been important in cancer treatment. ICI combination therapy has demonstrated broad therapeutic prospects for several solid cancers, including RCC (21–25). With the widespread use of ICI-based immunotherapy for RCC, the safety of ICI combination therapy is receiving increasing attention from clinicians.

To the best of our knowledge, this study is the first to perform a comprehensive evaluation of the severe and fatal toxicities of ICI combination therapy compared with standard VEGF-TKI–targeted therapy in patients with mRCC. The analysis showed that ICI combination therapy did not cause more SAEs or FAEs than TKI. Furthermore, the incidence of FAEs with ICI combination therapy was very low (1.2%). The most common FAEs were pneumonitis, hepatitis, hemorrhagic events, and myocarditis, which deserve attention in clinical practice. Wang et al. (26) evaluated the fatal toxic effects associated with ICIs in cancer, measuring the toxicity-related fatality rates for anti–PD-1, anti–PD-L1, anti–CTLA-4, and anti–PD-1/PD-L1 plus anti–CTLA-4 treatments as 0.36%, 0.38%, 1.08%, and 1.23%, respectively. Their findings supported the low incidence of FAEs in cancer patients treated with dual immunotherapy (PD-1/PD-L1 plus CTLA-4). Except for hypertension (18.6%), the incidence of SAEs with ICI combination therapy was low (<10%). These results suggest that ICI combination therapy has a good safety profile for the treatment of mRCC.

Considering the possible effect of different ICI combinations on SAEs, we conducted a subgroup analysis according to different therapeutic regimens and found that PD-L1 plus VEGF inhibitors had similar SAEs, and PD-1 plus CTLA-4 inhibitors had fewer SAEs than TKI. However, PD-1 plus VEGF inhibitors were associated with more SAEs than TKI alone. Similar results have been reported. For example, in the IMbrave150 trial, Finn et al. (27) reported that patients receiving atenizumab (anti–PD-L1) and bevacizumab had a similar risk of Grade 3 or 4 adverse events (56.5% vs. 55.1%, respectively) as those receiving sorafenib as the first-line treatment for unresectable hepatocellular carcinoma (HCC). In addition, Ren et al. (28) reported that sintilimab (anti–PD-1) plus bevacizumab biosimilar showed more SAEs (32% vs. 19%) than sorafenib in patients with unresectable HCC. This finding suggests that, despite ICI combination therapy having a good overall safety profile, some differences in toxicities among different ICI combinations may need to be treated differently.

In addition to severe toxicity overall, we also focused on the SAEs in each organ system. We found that ICI combination therapy had significantly less hematotoxicity, including anemia, neutropenia, and thrombocytopenia, than TKI. This result may indicate that ICIs have a protective effect on the blood system, thus reducing the damage to bone marrow cells caused by TKI-targeted agents. Kramer et al. (29) reported that hematological immune-related adverse events could affect all hematopoietic blood cell lineages and may persist or even be fatal; therefore, clinicians should monitor blood counts closely, if necessary. Although hematotoxicities are rare, we found that patients receiving ICI combination therapy showed greater hepatotoxicity (ALT and AST increases) than those receiving TKI; some died of immune-related hepatitis. Wang et al. (30) reported that using ICIs was associated with greater hepatotoxicity than all other systematic treatments and that ICI combination therapy had a greater risk of hepatotoxicity (including hepatic failure) than ICI monotherapy. Because of the significant association between ICI-based therapy and hepatotoxicity, clinicians should focus more attention on this safety signal. For nephrotoxicity, ICI combination therapy did not increase the risk of creatinine and renal failure but significantly increased the risk of proteinuria. Wu et al. (31) reported that adding ICI to VEGF inhibitors might cause hypertension and proteinuria. Ning et al. (32) reported that, after long-term targeted therapy, the use of combination therapy further aggravated proteinuria in patients with mRCC. In addition, ICI combination therapy showed a higher frequency of decreased appetite, increased lipase levels, and adrenal insufficiency than TKI; therefore, gastrointestinal toxicity and endocrine toxicity cannot be easily ignored when selecting ICI combination therapy for patients with mRCC. Therefore, although the overall safety was good, the toxicity of ICI combination therapy was found to have high organ specificity, which deserves attention in clinical practice. Another noteworthy aspect is that in this analysis, most included RCTs used sunitinib as a control drug. However, in current clinical practice, cabozantinib has become a preferred choice of monotherapy for patients with mRCC. So, whether our results are applicable to other TKI drugs, more detailed research is required.

This study had some limitations. First, only a few combined modes of ICI therapy are currently used to treat mRCC, including PD-1/PD-L1 plus VGFR inhibitors and PD-1 plus CTLA-4 inhibitors. No combination of ICI with chemotherapy was studied; therefore, toxicity results must be analyzed and generalized to other tumors carefully. Second, we found the results of each ICI-combination subgroup were different when we analyzed the SAEs of the patients. We point this out because different types of ICI, including inhibitors of PD-1/PD-L1 and CTLA-4, have different antitumor mechanisms, so each combination needs to be treated differently. Third, because clear cell RCC was the main inclusion criteria in all presented clinical trials, our results may not be extrapolated to another histology type of RCC. Finally, because of limited data, we could not conduct a subgroup analysis according to the different ICI combinations for each SAE, which may have affected the results of these toxicities.

## Conclusion

The current meta-analysis demonstrates that, compared with TKI, ICI combination therapy has a safe profile in general and does not increase SAEs and FAEs in patients with mRCC. Moreover, ICI combination therapy showed significantly less hematotoxicity than TKI. However, higher frequencies of SAEs were also observed, including hepatotoxicity, gastrointestinal toxicity, endocrine toxicity, and nephrotoxicity, warranting closer attention in clinical and future investigations.

## Data availability statement

The original contributions presented in the study are included in the article/supplementary material. Further inquiries can be directed to the corresponding author.

## Author contributions

Y-NF, G-YX, and LX designed the study. D-CM and J-FH screened the articles and extracted the relevant data. P-HL, G-YX, and X-JL contributed to the statistical analysis. Y-NF and LX wrote the manuscript. All authors contributed to the article and approved the submitted version.
